# USADAE: a deep learning approach to disentangle hidden covariates in RNA-seq data

**DOI:** 10.1093/bib/bbag261

**Published:** 2026-05-31

**Authors:** Xu Chen, Luoyuan Guo, Yaosheng Chen, Delin Mo, Xiaohong Liu

**Affiliations:** State Key Laboratory of Biocontrol, School of Life Sciences, Sun Yat-Sen University, No. 135, Xingang West Road, Haizhou District, Guangzhou, Guangdong 510275, China; State Key Laboratory of Biocontrol, School of Life Sciences, Sun Yat-Sen University, No. 135, Xingang West Road, Haizhou District, Guangzhou, Guangdong 510275, China; State Key Laboratory of Biocontrol, School of Life Sciences, Sun Yat-Sen University, No. 135, Xingang West Road, Haizhou District, Guangzhou, Guangdong 510275, China; State Key Laboratory of Biocontrol, School of Life Sciences, Sun Yat-Sen University, No. 135, Xingang West Road, Haizhou District, Guangzhou, Guangdong 510275, China; State Key Laboratory of Biocontrol, School of Life Sciences, Sun Yat-Sen University, No. 135, Xingang West Road, Haizhou District, Guangzhou, Guangdong 510275, China

**Keywords:** autoencoder, adversarial learning, confounder disentanglement, RNA-seq

## Abstract

Integrative analysis of RNA-seq datasets faces critical challenges in disentangling biologically meaningful signals from implicit confounders, such as unmeasured technical variability and nonlinear interactions between biological and technical variables. Traditional methods like Surrogate Variable Analysis (SVA), Probabilistic Estimation of Expression Residuals (PEER), and Remove Unwanted Variation (RUVSeq) rely on linear assumptions, which generally fail under complex nonlinear confounding patterns. Although deep learning approaches show promise in single-cell RNA-seq, they primarily address known batch labels rather than disentangling hidden confounders. Here, we develop a novel autoencoder framework coupled with adversarial learning, UnSupervised Adversarial Deconfounding AutoEncoder (USADAE), specifically designed to separate confounders from biological signals. The model encodes RNA-seq data into distinct biological and confounder latent spaces through adversarial disentanglement, enabling downstream correction of differential expression and eQTL analysis. In comprehensive simulations, USADAE significantly outperforms existing methods in extracting covariates while preserving biological signals. Real-data applications further demonstrate its robustness across diverse scenarios, including cancer genomics and eQTL studies.

## Introduction

In recent years, numerous RNA-seq datasets have been generated from different experimental conditions or laboratories [1–4]. However, the utility of integrative analysis depends on separating true biological signals from confounders. For instance, batch effects arising from technical variability in sequencing depth or library preparation can mimic true differential expression, thereby significantly impacting downstream analysis and leading to spurious associations and false positives [5–7]. Similarly, in eQTL (expression quantitative trait loci) studies, data affected by batch effects or unaccounted technical variation may result in spurious associations [8]. In some cases, explicit covariates can be obtained by recording known variables. However, implicit covariates that affect gene expression are often unmeasured or inherently unobservable for various reasons. Therefore, disentangling these implicit covariates from observed signals is important for subsequent differential expression analysis (DEA) or eQTL analysis.

Traditional confounder correction strategies in DEA are divided into supervised and unsupervised methods [9]. Supervised approaches, like Combat and Combat-seq, rely on known covariates [7, 10]. Unsupervised methods include Surrogate Variable Analysis (SVA), which assumes linear confounder effects, and Remove Unwanted Variation (RUV) methods like RUVG and RUVR [11]. Probabilistic Estimation of Expression Residuals (PEER), commonly used in eQTL studies [12–14], models gene expression as a combination of known covariates (biological signals) and latent factors (hidden covariates), estimated via a Bayesian framework [8]. Although these traditional methods have shown significant potential in disentangling unmeasured covariates, they generally assume that observed variables are linear combination of true biological signals and hidden covariates, less effective for nonlinear interactions common in real-word datasets [15, 16].

Recent studies demonstrated the capacity of deep learning to extract meaningful representations from RNA-seq data, especially in single-cell RNA-seq (scRNA-seq). Autoencoder-based frameworks like BERMUDA [17], scVI [18, 19], DESC [20], scANVI [21], and Fugue [22] employ reconstruction losses to learn low-dimensional embeddings that preserve cellular identity while correcting batch effects. deepMNN [23] abandons autoencoders for a residual network, explicitly using cross-batch PCA-space MNN pairs to guide correction. AD-AE [24] and cr-XVAE [16], designed for bulk RNA-seq, leverages known covariates information to remove confounders. While these methods are powerful, they are primarily applied to scRNA-seq data for tasks such as cell clustering in latent space or batch effect correction when batch labels are known. However, they often fail to explicitly disentangle hidden covariates, which could improve downstream analysis when covariates are unknown. Therefore, there is a need for a novel method capable of identifying complex implicit covariates for subsequent analysis. To address this problem, we introduced a deep-learning-based model named UnSupervised Adversarial Deconfounding AutoEncoder (USADAE), designed to disentangle biological signal and hidden covariates from RNA-seq data. USADAE employs two autoencoders to encode observed signals into distinct biological and confounder latent representations. Subsequently, an adversarial network is utilized to disentangle these biological and confounder latent spaces. Then, the extracted hidden covariates can be utilized in subsequent DEA or eQTL analysis to correct spurious associations. USADAE demonstrated better performance compared to competing methods in simulation studies and real data analysis across diverse downstream applications.

## Results

### Framework of USADAE algorithm

**Figure 1:**
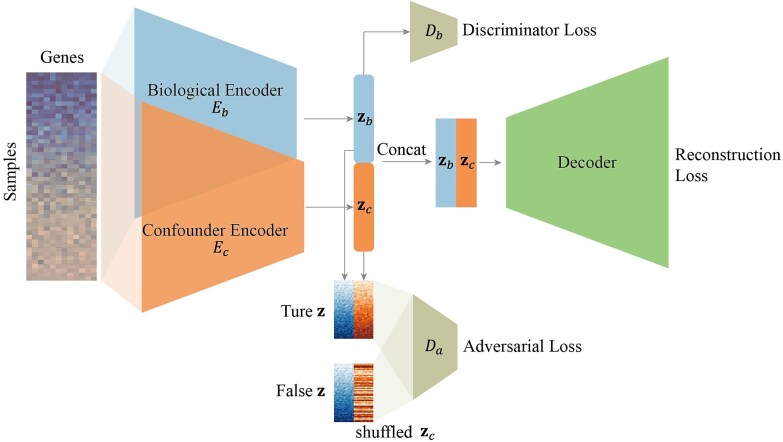
Sketch of USADAE framework. USADAE employs dual encoders to decompose RNA-seq data into biologically relevant (${\mathbf{z}}_b$) and confounder-associated (${\mathbf{z}}_c$) latent representations, concatenated for decoder-driven reconstruction. The autoencoder operates along the gene-expression feature dimension, reducing the dimensionality of expression profiles while preserving sample-wise relationships. An adversarial discriminator (${D}_a$) enforces statistical independence between ${\mathbf{z}}_b$ and ${\mathbf{z}}_c$ by distinguishing true $\left[{\mathbf{z}}_b,{\mathbf{z}}_c\right]$ from shuffled pairs, while a weak discriminator (${D}_b$) guides the biological encoder to retain label-discriminative features (e.g. case/control). Through adversarial optimization, USADAE explicitly isolates hidden covariates from observed signals.

USADAE is a model leveraging a deep learning strategy for the explicit disentanglement of hidden covariates from RNA-seq data. The model employs two encoders to transform the gene expression profile into distinct low-dimensional biological (${\mathbf{z}}_b$) and hidden confounder (${\mathbf{z}}_c$) latent representations. These two latent representations are subsequently concatenated as $\left[{\mathbf{z}}_b,{\mathbf{z}}_c\right]$ and fed as input to a decoder (Fig. 1).

To achieve the disentanglement of the biological and confounder latent spaces, an adversarial learning framework is utilized. Specifically, a discriminator, denoted as ${D}_a$, is introduced. This discriminator is trained to distinguish between “true” data, formed by concatenating the original biological and confounder latent ($\left[{\mathbf{z}}_b,{\mathbf{z}}_c\right]$) and “false” data generated by concatenating the biological latent with a shuffled version of the confounder latent ($\left[{\mathbf{z}}_b,\mathrm{shuffled}\left({\mathbf{z}}_c\right)\right]$). The encoders are simultaneously optimized to deceive ${D}_a$, thereby ensuring the statistical independence of the biological and confounder latent spaces.

Furthermore, to encourage the biological encoder to exclusively capture biological signals rather than confounder information, a weak discriminator, ${D}_b$, is additionally incorporated. This discriminator is designed to distinguish biological labels (e.g. control versus case groups) as effectively as possible.

Through the synergistic introduction of ${D}_a$ and ${D}_b$ discriminators and the application of adversarial learning, USADAE can explicitly disentangle biological and confounding signals within the latent space.

### Model evaluation on simulated differential expression data

**Figure 2:**
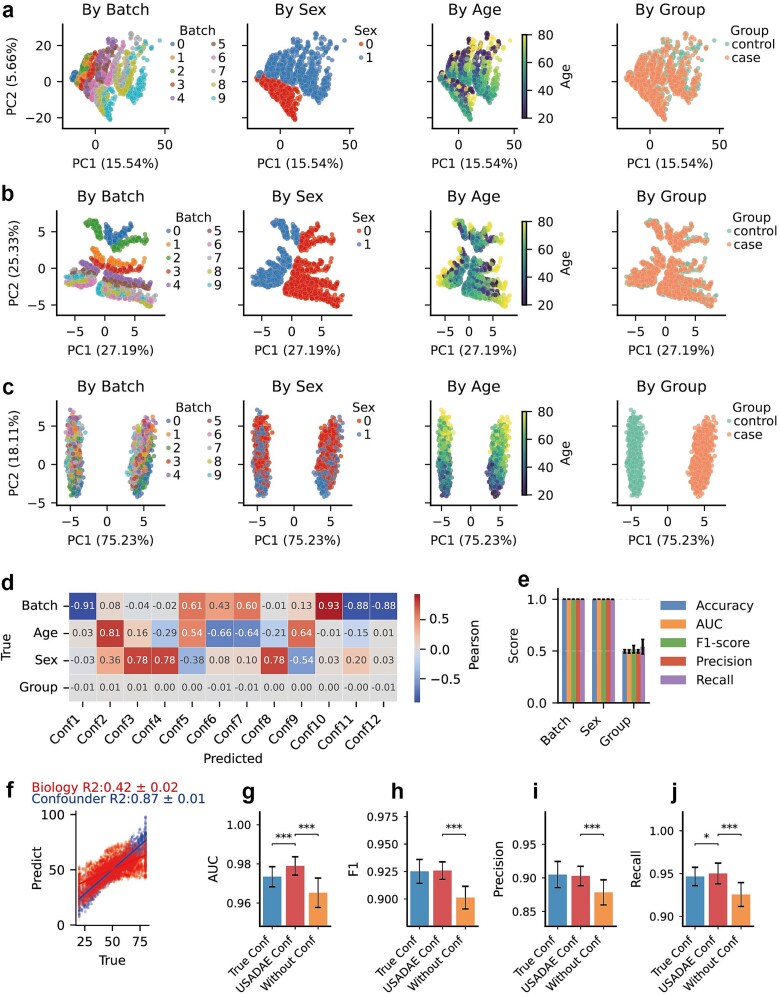
Performance evaluation of USADAE on simulated dataset. (a) PCA plot colored by batch, age, sex, and group, with PC1 and PC2 variance explained. (b, c) PCA plots of confounder and biological latent spaces respectively. (d) Correlation heatmap between USADAE’s covariates and true covariates and group. (e) Predict performance for batch, sex, and group using USADAE’s covariates across 5-fold cross-validation. (f) Scatter plot compares true and predicted age from confounder (blue) and biological latent spaces (red). (g–j) Evaluation of DEGs detection by different metrics. Error bars represent the standard deviation across 10 independent analyses; significance markers (paired t-tests and Benjamini–Hochberg adjusted, ^*^adjusted *P* < .05, ^**^adjusted *P* < .01, ^***^adjusted *P* < .001) indicate statistical differences between USADAE conf and other groups. AUC is based on full ROC curves computed across all *P*-value thresholds, while others use the threshold in Methods.

To evaluate our model’s ability to disentangle covariates, we simulated an RNA-seq dataset, with strong separation patterns driven by batch, sex, and age (Fig. 2a).

The USADAE’s covariates (Fig. 2b) maintained alignment with true covariates without exhibiting group-level separation. In the biological latent space, the separation related to true covariates was no longer apparent, while the separation between case and control groups became more distinct (Fig. 2c). The data corrected by USADAE show the random separation by covariates, while retaining the separation pattern similar to the pure biological signal before adding confounders (Supplementary Fig. S1).

Specific confounder dimensions (e.g. Conf1, Conf2, Conf3) strongly correlated with known covariates (Fig. 2d). To further assess the effectiveness of USADAE’s covariates, classification or regression models were developed using them. Predictive models achieved near-perfect for categorical covariates (Fig. 2e) and accurately reconstructed the continuous covariate age (R^2^ = 0.87 ± 0.01), significantly outperforming the biological latent space (R^2^ = 0.42 ± 0.02) (Fig. 2f). Moreover, the confounder space showed random-level prediction for the biological group, indicating minimal information leakage.

We performed 10 independent simulations using distinct random seeds. DEA incorporating USADAE’s covariates (USADAE Conf) yields significantly higher area under the curve (AUC), F1-score, precision, and recall than model without covariates (Fig. 2g–j). Notably, USADAE improved AUC over true covariates by capturing nonlinear effect that may distort *P*-value rankings, while others remained unchanged at fixed thresholds where true confounders already perform adequately. These findings indicate USADAE’s enhanced DEA accuracy through confounder separation.

### Comparative performance of USADAE in confounder separation and differential expression analyses

**Figure 3:**
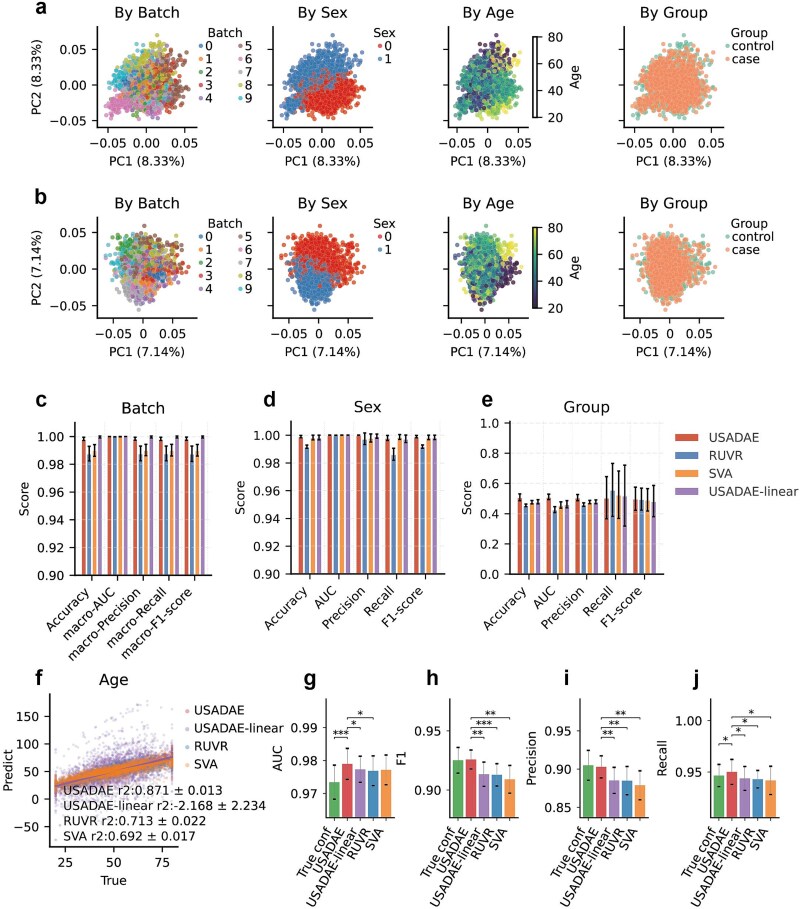
Performance comparison of USADAE, USADAE-linear, RUVR, and SVA for DEA on simulated dataset. (a, b) PCA plot of covariates estimated by RUVR and SVA, respectively. (c–e) Classification performance of logistic regression models using covariates from USADAE, RUVR, and SVA, averaged over 5-fold cross-validation with standard errors. (f) Mean R^2^ comparison of linear regression models using covariates from USADAE, USADAE-linear, RUVR, and SVA, averaged over 5-folds with standard errors. (g–j) Metrics comparison for DEA using covariates from USADAE, USADAE-linear, RUVR, SVA, and True Conf.

To evaluate how the USADAE algorithm performs relative to RUVR and SVA, a comparative analysis using simulated data was conducted. PCA plot revealed that the covariates estimated by RUVR (k = 12) and SVA exhibited weaker stratification patterns compared to those extracted by USADAE, in which latent embedding dimension was set to 12 (Figs 2b, 3a, and  b). Then, the effectiveness of confounder separation was evaluated by prediction score obtained from model constructed by estimated covariates.

USADAE and USADAE-linear show slightly higher performance than RUVR and SVA in batch and sex prediction (Fig. 3c–e). Here, USADAE-linear refers to a variant of USADAE in which activation functions are removed from the decoder, allowing us to assess whether USADAE effectively learns nonlinear confounding structure. For biological group, all performed at chance level, with USADAE showing lower variance. For age, USADAE’s covariates yielded R^2^ = 0.871 ± 0.01, surpassing RUVR (0.713 ± 0.02) and SVA (0.692 ± 0.02) (Fig. 3f, Supplementary Table S1), indicating more stable confounder capture.

To evaluate the significance, 10 independent analyses were conducted using the simulated data described above. USADAE achieved significantly higher score than others (Fig. 3g–j, Supplementary Fig. S1F). These results show the robustness of USADAE in isolating covariates while preserving biological signals.

To evaluate the computational efficiency, we compared memory usage and runtime on simulated datasets with varying sample sizes. USADAE demonstrated superior computational efficiency, with substantially shorter runtime and lower memory usage than RUVR and SVA, particularly at larger sample sizes (Supplementary Fig. S2A and B).

### Validation of USADAE performance on real breast cancer datasets

To validate the performance of the USADAE on real-world data, we applied it to breast cancer datasets (GSE2034 [25], GSE7390 [26], GSE20194 [27, 28], GSE25066 [29], GSE62725 [30], and GSE20271 [31]), which exhibit diverse technical and biological variability.

**Figure 4:**
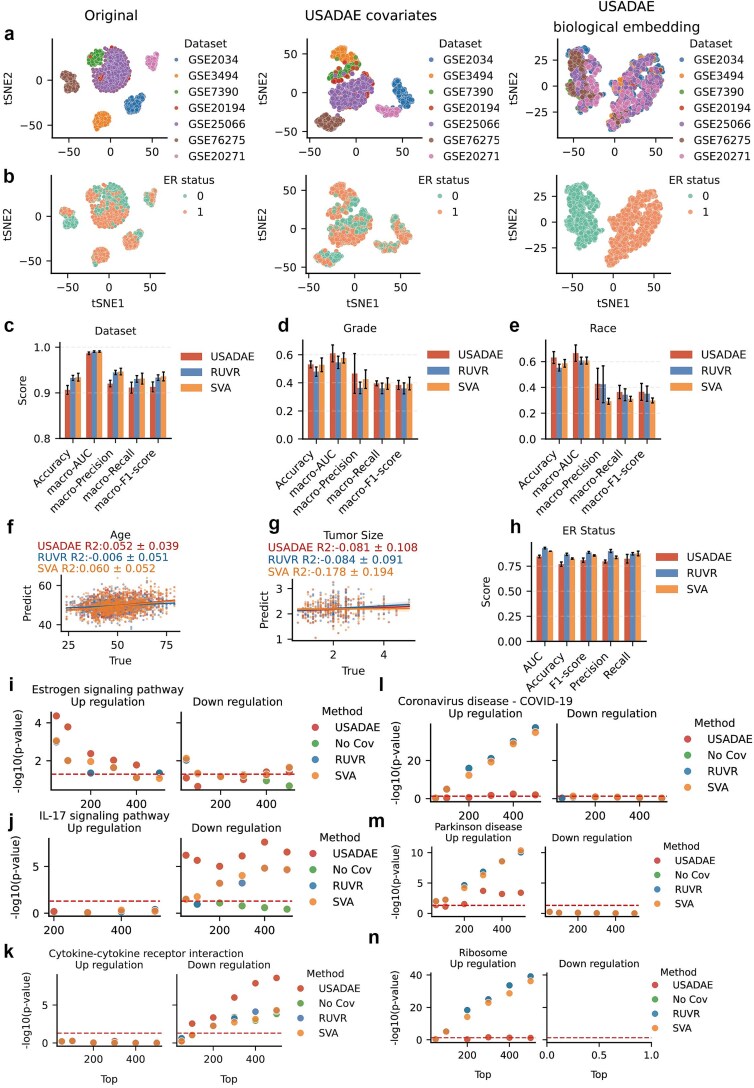
Performance comparison of USADAE, RUVR, and SVA on breast cancer datasets. (a) t-SNE plot colored by dataset. From left to right, the panels display: (1) the original data, (2) USADAE’s covariates, and (3) USADAE’s biological latent. (b) t-SNE plot, similar to (a), colored by ER status. (c–e) Mean classification performance (dataset, grade, race) of random forest models using covariates from USADAE, RUVR, SVA, averaged over 5-fold cross-validation, with standard error bars. (f, g) Scatter plots of predicted and true covariates, showing mean R^2^ with standard errors. (h) Bar charts comparing random forest performance for ER status. (i–n) Scatter plots of -log10 (*P*-value) for pathway enrichment of top differentially expressed genes, with (i–k) ER-related and (l–n) non-ER-related pathways.

T-SNE projections of gene expression data showed distinct dataset-specific clusters, with no clear separation by estrogen receptor (ER) status (Fig. 4a and b, left). For USADAE’s covariates, dataset-specific clustering persisted, capturing technical confounders without encoding ER status (Fig. 4a and b, middle). Conversely, the biological latent space reduced dataset-specific clustering and revealed clear ER+ versus ER- separation (Fig. 4a and b, right), demonstrating USADAE’s ability to suppress confounders while amplifying biological signals.

To evaluate the accuracy of different algorithms in separating covariates, logistic regression and random forest classification models were constructed using covariates estimated by various methods for dataset, grade, and race, while linear and random forest regression models were developed for age and tumor size (Supplementary Table S2). USADAE outperformed RUVR and SVA in predicting grade and race, showed comparable performance for dataset, age, and tumor size (Fig. 4c–g), but had lower performance for ER status, indicating minimal biological information in its confounder estimates (Fig. 4h).

Since true DEGs are unknown in real data, the performance of confounder separation algorithms was evaluated by examining the relevance of enriched pathways to known biological processes. DEA incorporating covariates from USADAE, RUVR, and SVA was conducted, with adjusted thresholds to ensure comparable DEG counts (FDR < 0.05, |FC| > 1.2 for USADAE; FC > 1.5 or < −1.2 for others) (Supplementary Fig. S3A–D). Notably, Kyoto Encyclopedia of Genes and Genomes (KEGG) pathway enrichment analysis revealed that RUVR, SVA, and “no covariate” methods enriched irrelevant pathways (e.g. “Coronavirus disease - COVID-19”), while USADAE avoided such enrichments and significantly enriched breast cancer-relevant pathways like IL-17 signaling [32] (Supplementary Fig. S3E and F). To further evaluate the biological relevance and specificity of USADAE, enrichment analysis was conducted using the top up-regulated or down-regulated 50–500 DEGs derived from ER+/ER- breast cancer-related pathways (estrogen signaling [33, 34], IL-17 signaling, cytokine–cytokine receptor interaction [35, 36]) and unrelated pathways (e.g. Parkinson’s disease, COVID-19). USADAE consistently yielded lower *P*-values for breast cancer pathways, particularly for upregulated genes in estrogen signaling (ER+ specific) and downregulated genes in IL-17 signaling and cytokine interactions (ER- specific) (Fig. 4i–k, Supplementary Fig. S4). For unrelated pathways, USADAE showed higher *P*-values, indicating greater specificity by avoiding spurious enrichments (Fig. 4l–n). To evaluate whether USADAE captures nonlinear confounding structure beyond linear model, we compared the biological embeddings of USADAE and USADAE-linear. Under PCA, a linear dimensionality reduction method, both models separated samples by ER status but not by dataset (Supplementary Fig. S5A and B, left and middle). Under t-SNE, a nonlinear embedding method, USADAE-linear exhibited dataset-specific clustering similar to SVA and RUVR (Supplementary Fig. S5A–D), a pattern absent in USADAE (Fig. 4a and b, right). Accordingly, differential expression results with USADAE-linear covariates resembled those from linear methods (Supplementary Fig. S5E and F).

### Application of USADAE in eQTL analysis with simulated gene expression data

To evaluate the utility of the USADAE algorithm in eQTL analysis, we simulated gene expression data across different tissues using real genotype data. USADAE was applied to extract covariates for eQTL analysis, compared to PEER, PCA [37], and Control (covariates = 1).

**Figure 5:**
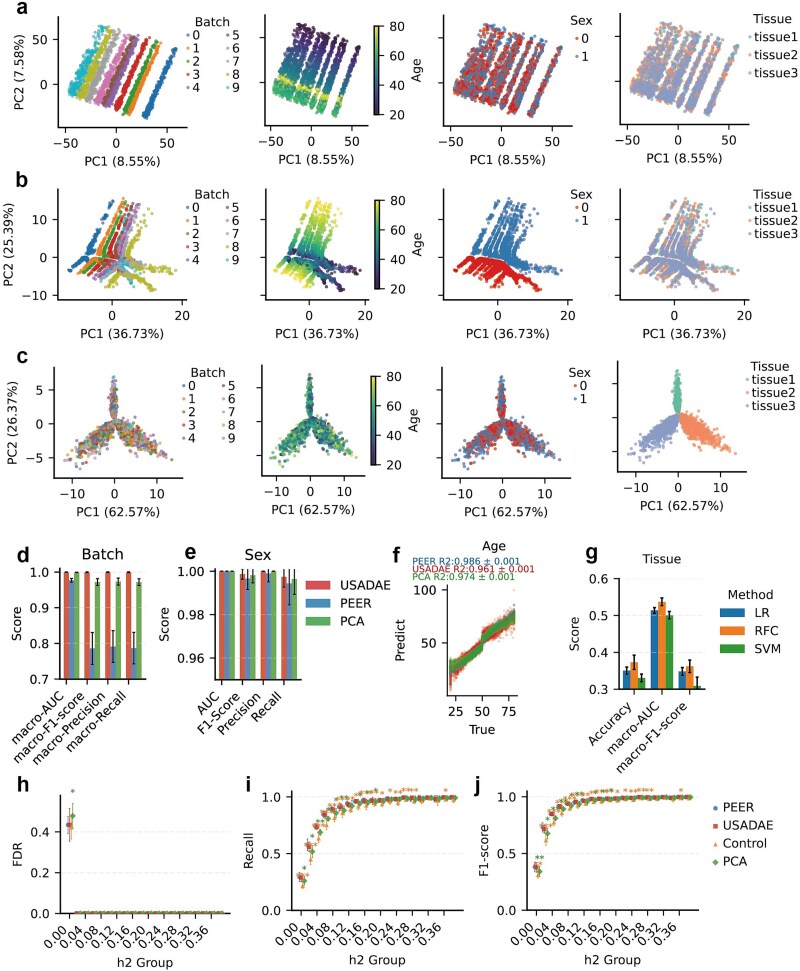
Performance comparison of USADAE and PEER in eQTL analysis with simulated gene expression data. (a) PCA plot colored by batch, age, sex, and tissue. (b, c) PCA plots for USADAE’s covariates and biological latent respectively. (d, e) Bar charts comparing random forest classification performance for batch and sex using USADAE and PEER covariates, averaged over 5-fold cross-validation with error bars. (f) Scatter plots of predict and true age values, showing mean R^2^ with standard errors. (g) Predict performance for tissue of different model constructed using covariates estimated by USADAE. LR, logistic regression; RF, random forest classifier; SVM, support vector machine classifier. (h–j) Scatter plots evaluate eGene detection performance across h^2^ groups using USADAE, PEER, PCA covariates, and control (covariate = 1). Significance markers (paired t-tests with Benjamini–Hochberg adjustment, ^*^adjusted *P* < .05, ^**^adjusted *P* < .01) represent differences between USADAE Conf and other groups: yellow asterisks for USADAE versus control, blue for USADAE versus PEER (no significance observed).

PCA plot showed that samples clustered by batch, sex, and age, but not tissue (Fig. 5a). In USADAE’s confounder latent space, clustering by true covariates remained evident (Fig. 5b). In the biological latent space and corrected data, confounder clustering disappeared, revealing distinct tissue-type clustering aligned with intrinsic biological signals (Fig. 5c, Supplementary Fig. S6A and B). In PEER’s covariates, sex and age clustering remained, but batch effects were less evident (Supplementary Fig. S6C and D). For confounder prediction, USADAE’s covariates outperformed PEER in predicting batch and sex, with comparable age prediction and tissue prediction (Fig. 5d–g). These indicate that the confounder latent space successfully concentrates non-biological variation, which can be used as covariates in downstream eQTL analysis. Across 10 independent eQTL analyses, USADAE demonstrated similar performance compared to PEER, while showing significantly higher recall and F1-score than the PCA and Control at low h^2^ (Fig. 5h–j). On the simulated dataset, USADAE showed significantly shorter cumulative runtime than PEER across three tissues, while PEER required less memory for single-tissue analysis (Supplementary Fig. S2C–E).

### Application of USADAE in eQTL analysis with PigGTEx datasets

To assess the performance of the USADAE algorithm in real-world eQTL analysis, it was applied to PigGTEx datasets, focusing on evaluating confounder removal, stability across data splits, and *cis*-heritability (cis-h^2^) of eGenes, compared to PEER (optimal factor number = 10), PCA (optimal PC number = 18) and Control methods.

**Figure 6:**
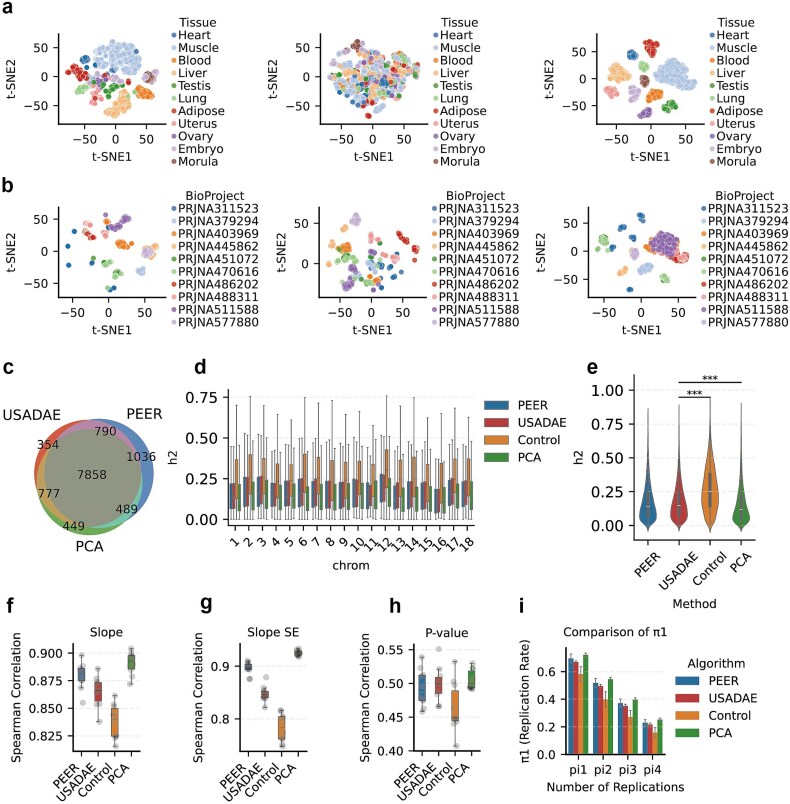
Performance comparison of USADAE, PCA, and PEER in eQTL analysis with PigGTEx datasets. (a) t-SNE plot colored by tissue. From left to right, the panels display: (1) the original data, (2) USADAE’s covariates, and (3) USADAE’s biological latent. (b) t-SNE plot, similar to (a), colored by BioProject. (c) Venn plot of eGenes. (d) Boxplot compares cis-h^2^ values of eGenes detected using covariates estimated by different methods. (e) Comparison of cis-h^2^ distribution of all eGenes. USADAE and PEER have similar distributions (*P* = .176, Wilcoxon test). (f–h) Boxplots display Spearman correlations for (f) slope estimates, (g) slope standard errors, and (h) *P*-values of eGene-eQTL pairs across folds. (i) Bar plots show eGene-eQTL pair replication rates across folds, with error bars indicating standard errors from-fold cross-validation.

The t-SNE plot of the original data revealed partial clustering by tissues, with testis, ovary, embryo, and morula proving challenging to distinguish (Fig. 6a, left), while identical tissues (e.g. muscle) from different BioProject (PRJNA486202, PRJNA488311, PRJNA511588) clustered together according to their BioProject origins (Fig. 6b, left). In USADAE’s confounder latent space, BioProject clustering improved, while tissue clustering became more random (Fig. 6a and b, middle). In the biological latent space, BioProject clustering diminished (e.g. muscle), and tissue separation increased (Fig. 6a and b, right).

DEA was conducted between muscle and adipose, as well as between muscle and heart (cardiac muscle), with covariates estimated by different algorithms. KEGG pathway analysis from muscle versus adipose showed consistent enrichment across methods (Supplementary Fig. S7A–C), likely due to significant biological differences outweighing technical confounders (Fig. 6a). For the more similar muscle versus heart comparison, USADAE reduced false-positive findings. Notably, pathways unrelated to muscle or heart biology—such as pancreatic secretion and bile secretion—were substantially suppressed (Supplementary Fig. S7D–F).

To evaluate the effectiveness of the extracted covariates, classification models were constructed for categorical covariates including sex, library layout, bio-project, breed group, breed, and sequencing model, while regression models were developed for continuous covariates such as age, clean reads, mapped reads, and mapping rate, consistent with the methodology described in the simulated data results. Classification models using USADAE’s covariates outperformed PEER for categorical variables, and regression models showed greater stability for continuous variables (Supplementary Table S3).

To evaluate the consistency of eGene detection, a comparative analysis was performed in muscle tissue between eGenes identified using USADAE’s covariates and PEER’s covariates as covariates. USADAE detected 9779 eGenes in muscle, of which 8555 overlapped with PEER (9838 eGenes). USADAE-specific eGenes enriched more muscle-related pathways (e.g. calcium signaling), unlike PEER’s less relevant pathways (Supplementary Fig. S8). Additionally, cis-h^2^ of eGenes across chromosomes were calculated to assess the proportion of expression variance explained by local genetic variants after confounder correction. USADAE showed similar cis-h^2^ values compared to PEER, but significantly lower cis-h^2^ values than Control (Fig. 6d and e). To assess stability, the dataset was separated into five equal parts using k-fold strategy and eQTL analysis was performed on each subset. USADAE showed slightly lower slope and slope SE correlations than PEER but outperformed Control, with comparable *P*-value correlations (Fig. 6f–h). The π replication rate was comparable to PEER but higher than Control (Fig. 6i), demonstrating USADAE’s robustness in reproducible eQTL detection across splits.

## Discussion

In summary, we propose a deep learning-based algorithm that explicitly extracts covariates from observed RNA-seq signals by encoding them into distinct biologically relevant and confounding latent representations via adversarial learning. It outperforms traditional methods like RUVR, SVA, and PEER by capturing nonlinear confounding structures, offering precise correction and stable performance. In breast cancer, USADAE enhances ER-related pathway enrichment, suggesting potential for biomarker discovery. In PigGTEx, it matches PEER’s performance while enabling multi-tissue confounder extraction, facilitating integrative analysis.

USADAE’s disentanglement capability, which relies on enforcing statistical independence in the latent space, requires large sample sizes. This constraint, shared by many deep learning models, confines its optimal use to large RNA-seq or integrated multi-source datasets. A recent study showed that PCA outperforms several methods under the additive model assumption: expression = genotype effect + covariate effect + noise [37]. However, its performance is limited in the presence of interactions between covariates and genotype effects (Supplementary Fig. S9). Furthermore, extending USADAE to jointly model genomic and transcriptomic variation represents a promising direction that may further improve the resolution and accuracy of latent covariate extraction.

Finally, dimensionality reduction via k-means++ is currently used to mitigate overfitting caused by high input dimensionality, but this inevitably introduces some information loss (Supplementary Fig. S10). As larger RNA-seq and multi-omics datasets become increasingly available, such preprocessing may no longer be necessary.

## Method

### Model architecture

We proposed a USADAE model to disentangle covariates from biological signals in observed data with unknown confounders. We assumed that the observed data are generated by a combination of biological signals and latent covariates. Based on this, we introduced two parallel encoders: a biological encoder ${E}_b$ consisting of fully connected layers (${D}\rightarrow{512} \rightarrow{256} \rightarrow{L}$) with batch normalization and ReLU activations, producing a latent representation ${\mathbf{z}}_b$, and a confounder encoder ${E}_c$ (${128}\rightarrow{64} \rightarrow{L}_c$) that captures hidden covariates ${\mathbf{z}}_c$. Here, *D* denotes the input dimension, *L* represents the dimensionality of the biological latent space, and ${L}_c$ indicates the dimensionality of the hidden covariate space. These representations are jointly decoded through $Decoder\left(\left[{\mathbf{z}}_b;{\mathbf{z}}_c\right]\right)$, composed of symmetric fully connected layers (*L* + ${L}_c \rightarrow{256}\rightarrow{512}\rightarrow{D}$), to reconstruct the input. The reconstruction loss is defined as:



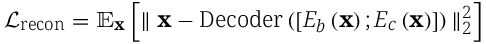



To enforce statistically independent representations of biological factors and hidden covariates, we implemented an adversarial disentanglement framework [38]. Specifically, we introduced a discriminator network ${D}_a$  $(({L} + {L}_c) \rightarrow{32} \rightarrow{32} \rightarrow{1})$ that learns to distinguish between two embedding types: joint embeddings ${\mathbf{z}}_{\mathrm{joint}}=\left[{E}_b\left(\mathbf{x}\right);{E}_c\left(\mathbf{x}\right)\right]$ generated from the same sample, representing the natural co-occurrence of biological signals and hidden covariates [39, 40]. Shuffled embeddings ${\mathbf{z}}_{\mathrm{shuffled}}=\left[{E}_b\left({\mathbf{x}}^{(i)}\right);{E}_c\left({\mathbf{x}}^{(j)}\right)\right]$ for $i\ne j$ constructed by pairing biological embeddings with randomly permuted confounder embeddings, where ${\mathbf{x}}^{(i)}$ and ${\mathbf{x}}^{(j)}$ represent input sample $i$ and $j$, respectively.

The adversarial training follows a minimax objective [38]:



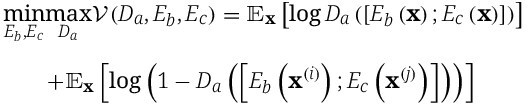



Through this adversarial formulation, the model enforces the statistical independence condition:


$$P\left({E}_b\left(\mathbf{x}\right),{E}_c\left(\mathbf{x}\right)\right)\to P\left({E}_b\left(\mathbf{x}\right)\right)P\left({E}_c\left(\mathbf{x}\right)\right)$$

thus, minimizing the Jensen–Shannon divergence between the joint distribution and the product of marginal distributions [41].

To ensure that ${E}_b$ specifically captures biological signals but not others, we added a weak discriminator ${D}_b$ that guides the biological encoder to retain label-discriminative features. The tissue discriminator loss is defined as:



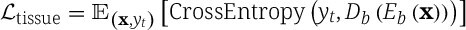



where ${y}_t$ represents the true tissue-type or condition label (e.g. ER status in breast cancer datasets or tissue type in PigGTEx). Finally, by combining these three synergistic objectives, the model optimization can achieve biologically meaningful disentanglement. The model training consists of three stages: (i) Autoencoder training, (ii) Autoencoder training + adversarial training, and (iii) Autoencoder training + adversarial training + tissue discrimination training. The epochs for stages 1 and 2 are set to 100, while the epochs for stage 3 are set to 500. Dropout layers are applied at the bottleneck layer of the model, and dropout rate is set to 0.4 by default [42]. The activation function used throughout the model is ReLU. Adam is employed across all training phases, with learning rates set to $1e-3$ for autoencoder training, and $1e-4$ for both adversarial and tissue discrimination training. The main tunable parameters in USADAE include the latent embedding dimension (*L*), the dropout rate, the λ values for adversarial training, and the number of centroids used in the preliminary k-means++ clustering step. In practice, we recommend setting *L* between 12 and 32, dropout between 0.2 and 0.6, ${\lambda}_{\mathrm{adv}}$ for adversarial between 0.5 and 2, ${\lambda}_{\mathrm{tissue}}$ for tissue equal to ${\lambda}_{\mathrm{adv}}$ plus 0.5, and the number of k-means++ centroids between 500 and 2500, depending on dataset size and signal complexity. All neural network models were implemented in PyTorch and trained on an NVIDIA GeForce RTX 3090 GPU with 24 GB memory.

### Simulated data for differential expression analyses

To assess the performance of USADAE in separating covariates, we simulated RNA-seq data comprising 3000 genes across 1000 samples (500 cases, 500 controls). Gene expression was influenced by three confounders-batch, age, and sex. Genes with |${\log}_2 FC$ | > 0.263 before adding confounders were treated as true significant differential expression genes (Supplementary Information).

### GEO datasets

We utilized seven breast cancer datasets from the GEO database (GSE2034, GSE3494, GSE7390, GSE20194, GSE20271, GSE25066, and GSE76275), comprising a total of 1544 samples with 12 548 genes. These datasets all contained ER status labels, which served as biological groups for downstream DEA.

To validate confounder accuracy, subsets with clinical annotations were extracted: 198, 268, and 502 samples with age from GSE7390, GSE20194, and GSE25066; 198 samples with tumor size from GSE7390; 198 and 502 samples with tumor grade from GSE7390 and GSE25066; and 265 samples with race from GSE20194. These were used to assess disentangled covariates, not included in training.

Log2-transformed data were processed with k-means++ clustering [43] to reduce dimensionality and prevent overfitting.

### Simulated data for eQTL analysis

We simulated multi-tissue eQTL data using PigGTEx chromosome 1 muscle genotypes, modeling 2000 genes across 3 tissues with tissue-specific, shared, and unique eGenes. *Cis*-regulatory effects used 5–15 SNPs per gene within 1 Mb, with effects scaled by MAF and heritability (0.5%–80%). Nonlinear confounders included batch, age, and sex effects. Expression combined genetic and confounding effects with tissue-specific noise. Genotype data included 10 000 SNPs (MAF > 1%) and replicated IDs for paired designs. Details are in the Supplementary Information.

### PigGTEx datasets

To evaluate the capability of USADAE in disentangling covariates across multi-group datasets, we integrated multi-tissue transcriptomic and genotypic data from PigGTEx project [13]. We analyzed 11 tissues (sample size >150 each), including muscle, adipose, and others. Transcriptomic data were preprocessed by dual-threshold filtering (TPM > 0.1 and raw count >6 in ≥20% samples per tissue), from which the top 10 000 high-variance genes were selected and aggregated across tissues. Prior to modeling, data were log2-transformed and dimension-reduced to 1500 via k-means++ clustering [43]. Genotype data and PCA covariates for *cis*-eQTL mapping were sourced directly from the PigGTEx database, retaining only SNPs with MAF ≥ 5%.

### Covariates evaluation

The accuracy of extracted covariates was evaluated by training machine learning models to predict known covariates withheld during training. For continuous and categorical variables, we employed regression and classification models, respectively, and assessed performance using a 5-fold cross-validation protocol. Model generalizability was quantified by R^2^ for regression tasks, and by AUC, F1-score, precision, and recall for classification tasks against the ground-truth labels (Supplementary Information).

### Differential expression analyses and evaluation

DEA were conducted using DESeq2 [44] for simulated data, a limma-voom [45] pipeline for public GEO datasets, with covariates (e.g. batch, sex, or those extracted by USADAE) integrated into the models. For simulated data, algorithm performance was evaluated by comparing predicted differentially expressed genes (DEGs) against a ground-truth list using ROC curves, with AUC measuring classification accuracy. True positives, false positives, false negatives, and true negatives were defined based on dual-threshold (adjusted *P*-values <.05 and |log2FC| > 0.263) and ground-truth status. Precision, recall, and F1-score assessed DEA improvement with covariates integration. For GEO datasets, where true DEGs are unknown, KEGG enrichment analysis on predicted DEGs was performed using clusterProfiler’s enrichKEGG functions [46].

### *Cis*-eQTL mapping

Similar to the PEER and PCA algorithm, USADAE was applied solely to RNA-seq data to infer hidden covariates. The model utilized the complete set of RNA-seq data from all tissues as input, with tissue identity serving as the grouping variable. Subsequently, the inferred covariates were applied as adjustments in *cis*-eQLT mapping to control for hidden confounders for each tissue. The analytical workflow in this analysis closely mirrors the MolQTL mapping approach outlined in the PigGTEx [13]. Key software or packages utilized include edgeR [47] and TensorQTL (1.0.10) [48].

### Replication rate (π) analysis

To assess the robustness of eQTL discoveries across methods, we computed replication rates (π statistics) via 5-fold cross-validation. In each fold, one subset served as the discovery set to identify significant eGene-eQTL pairs, while the remaining four were replication sets. The replication rate for a given discovery set was calculated as the proportion of its significant signals replicated in at least k (ranging from 1 to 4) of the replication datasets:


$${\pi}_k=\frac{\mathrm{Number}\ \mathrm{of}\ \mathrm{signals}\ \mathrm{replicated}\ \mathrm{at}\ \mathrm{least}\ k\ \mathrm{times}\ \left(\mathrm{n}\right)}{\mathrm{Total}\ \mathrm{number}\ \mathrm{of}\ \mathrm{signals}\ \mathrm{in}\ \mathrm{the}\ \mathrm{main}\ \mathrm{dataset}\ \left(\mathrm{N}\right)}$$

**Key Points**

- USADAE overcomes the limitations of linear methods by capturing nonlinear confounding patterns in RNA-seq data.
- USADAE optimizes Jensen–Shannon divergence to encode data into statistically independent biological and confounder latent spaces.
- USADAE robustly corrects covariates, enhancing differential expression and eQTL analyses with better performance across simulated and real-world datasets.

## Supplementary Material

Supplementary_naterials_bbag261

## Data Availability

Publicly available resources include: (i) the GEO datasets from the NCBI Gene Expression Omnibus (https://www.ncbi.nlm.nih.gov/geo/) and (ii) the RNA-seq data from the PigGTEx datasets (https://piggtex.farmgtex.org/). The code for the USADAE algorithm is available at https://github.com/chenxuya/USADAE.
