# Supplementary material for: USADAE: a deep learning approach to disentangle hidden covariates in RNA-seq data
Source: Brief Bioinform. 2026 May 31;27(3):bbag261. doi: 10.1093/bib/bbag261 (PMC13222517; doi:10.1093/bib/bbag261)
Supplement: Supplementary_naterials_bbag261 [file supplementary_naterials_bbag261.zip › Supplementary_naterials_bbag261.docx]

# Supplementary Figure

Supplementary figure 1


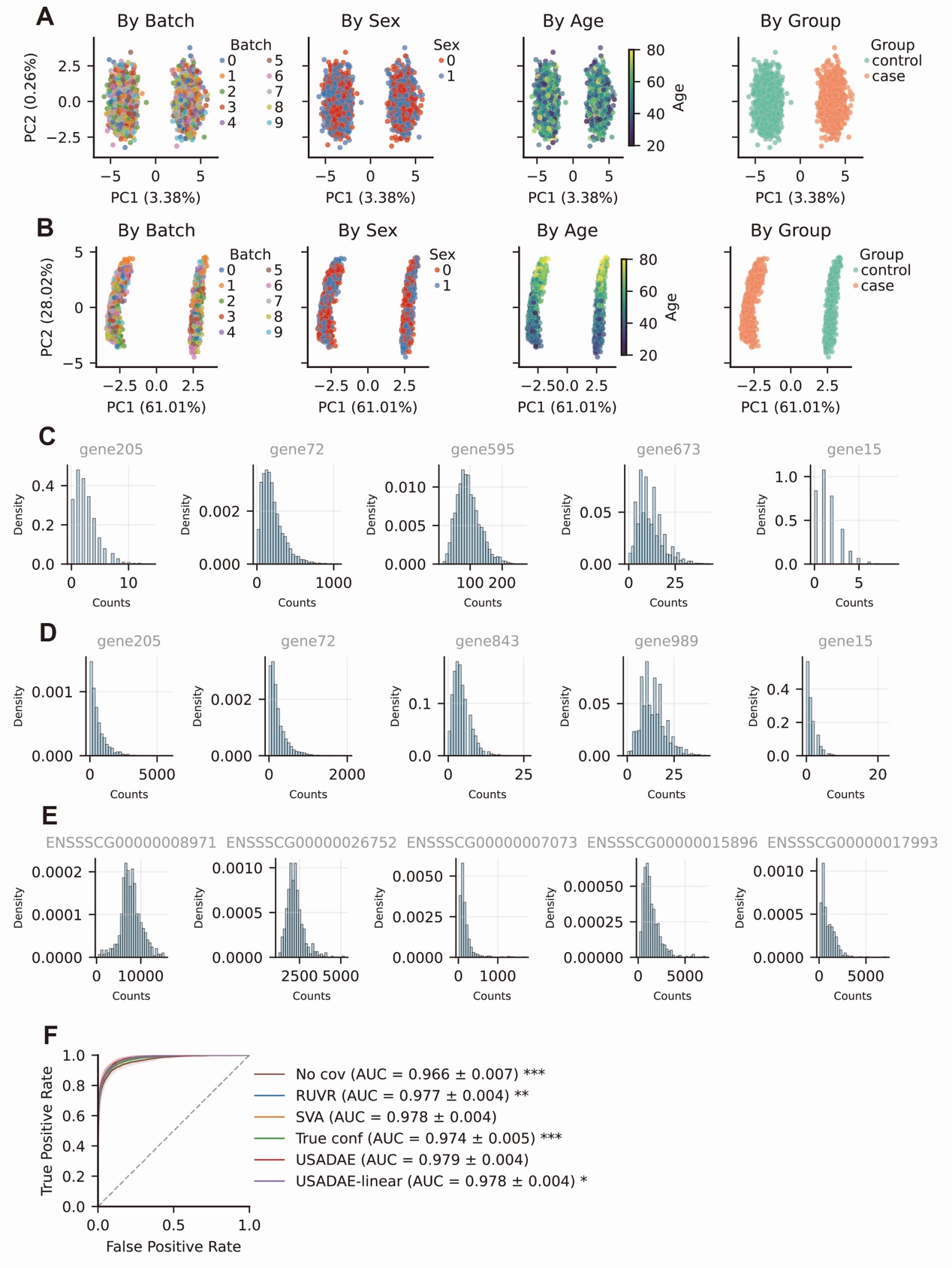


**(A, B)** PCA plot of biological signal before adding confounders and corrected data by USADAE. **(C, D, E)** Random selected gene expression distributions from simulated data (C, D) and empirical Pig GTEx muscle data (E). The high concordance between the confounder-incorporated distributions (D) and actual biological data (E) demonstrates the biological plausibility of our simulation approach. (F) ROC curves constructed with p-values from DEG after incorporating covariates from different algorithms. Significance markers (paired t-test with Benjamini-Hochberg adjusted, *p.adj < 0.05, **p.adj < 0.01, ***p.adj < 0.001) indicate statistically significant differences in AUC between USADAE and other methods.

**Supplementary figure 2**


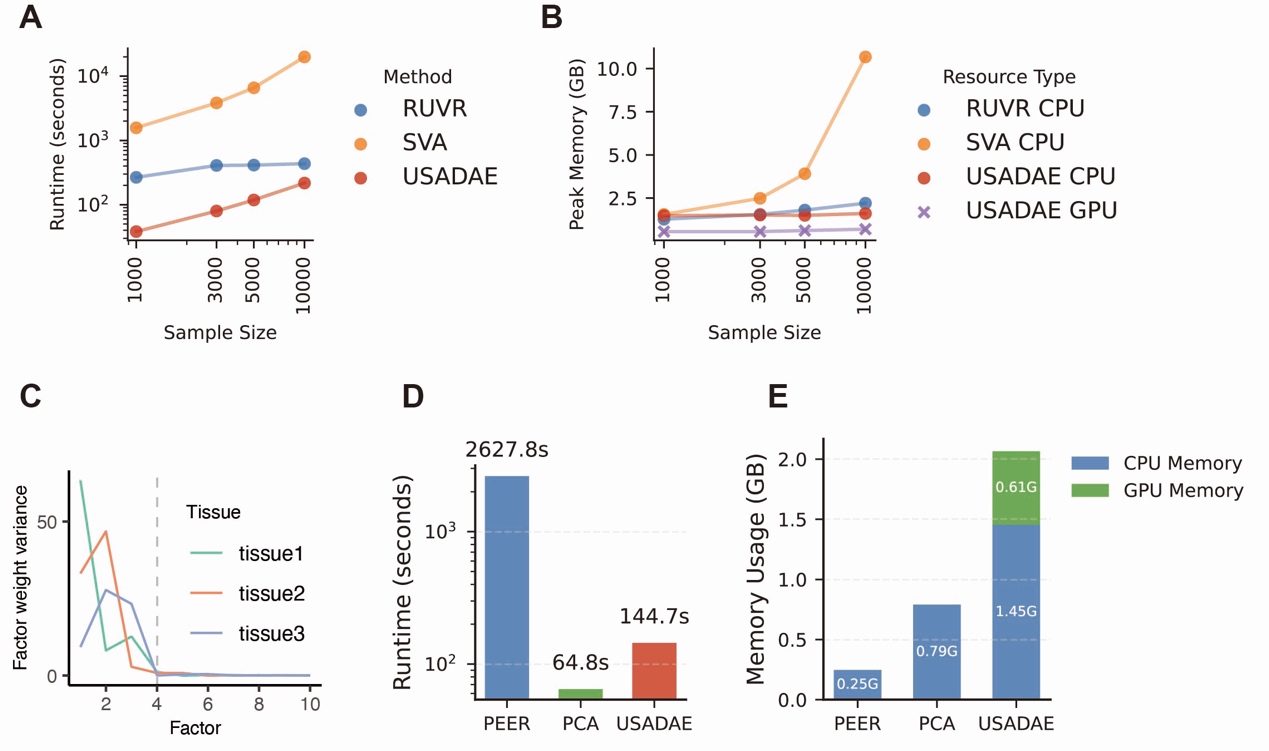


(A) Runtime of different algorithms across varying sample sizes. Batch size of USADAE was set to 1000 and epochs for stages 1, 2, 3 was set to 100, 100, and 500 respectively (B) Computational memory usage of different algorithms across varying sample sizes. (C) Selection of the optimal number of PEER factors. After identifying the optimal factor number, which was determined to be 4 in this simulated data, the PEER algorithm is rerun using this selected value. (D) Runtime comparison between PEER (with optimal factor number), PCA and USADAE on simulated datasets. (E) Comparison of computational memory usage between PEER, PCA and USADAE.

Supplementary figure 3


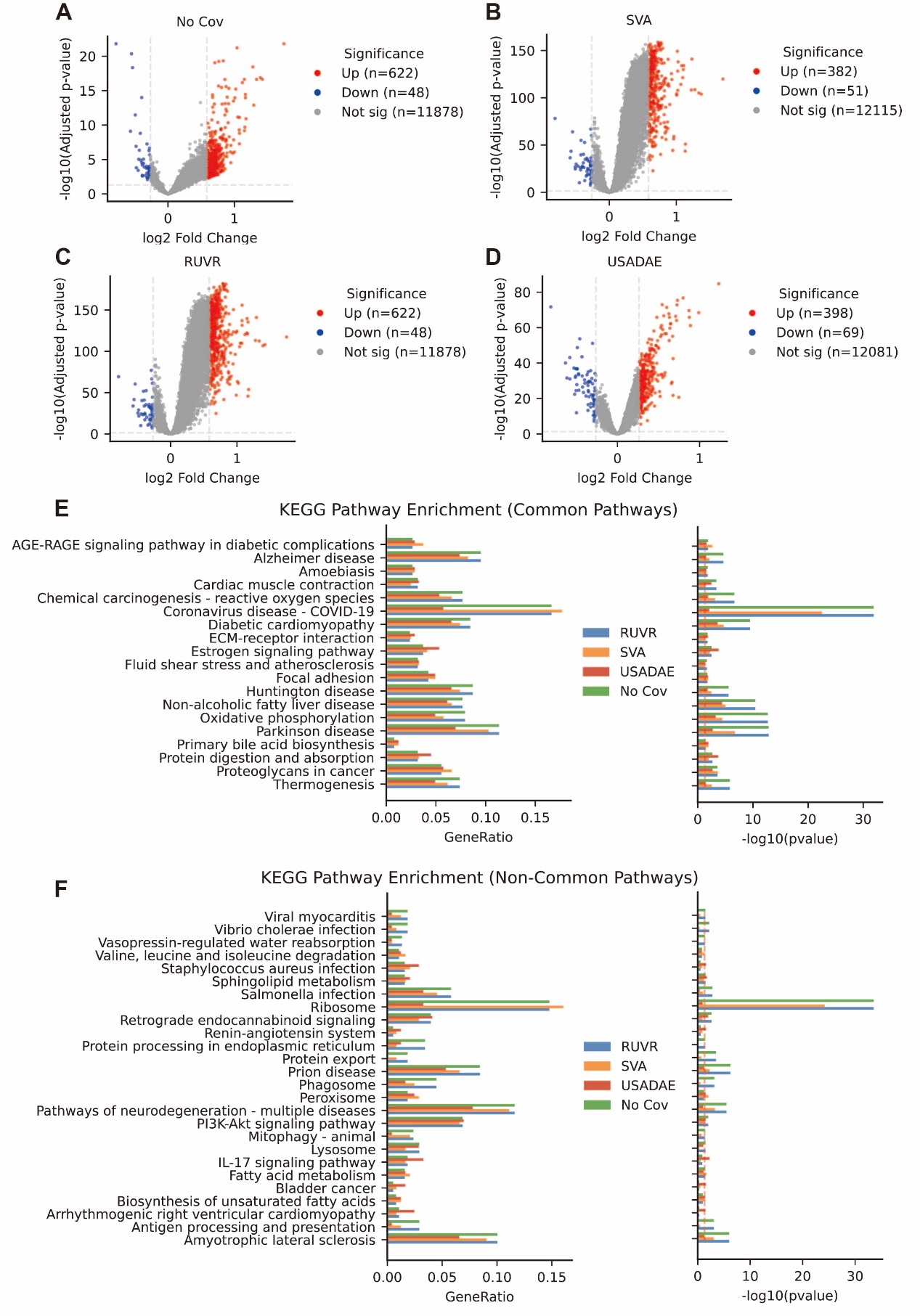


**(A, B, C, D)** Volcano plot of differentially expressed genes between ER+ and ER- breast cancer when using confounders estimated by No Cov, SVA, RUVR and USADAE as covariates respectively. x-axis represents log2 fold change, y-axis represents -log10(adjusted p-value). The horizontal dashed line represents adjusted p-value threshold of 0.05, while vertical dashed line represents log2 fold change threshold (No Cov, SVA and RUVR FC>1.5 or FC < -1.2; USADAE |FC|>1.2) **(E)** KEGG enrichment bar plot of differentially expressed genes detected above. Pathways significantly enriched in all methods are shown. **(F)** KEGG enrichment bar plot of differentially expressed genes detected above. Pathways significantly enriched at least in one method are shown. The x-axis of left panel represents the gene ratio, while the right panel represents the -log10(p-value).

Supplementary figure 4


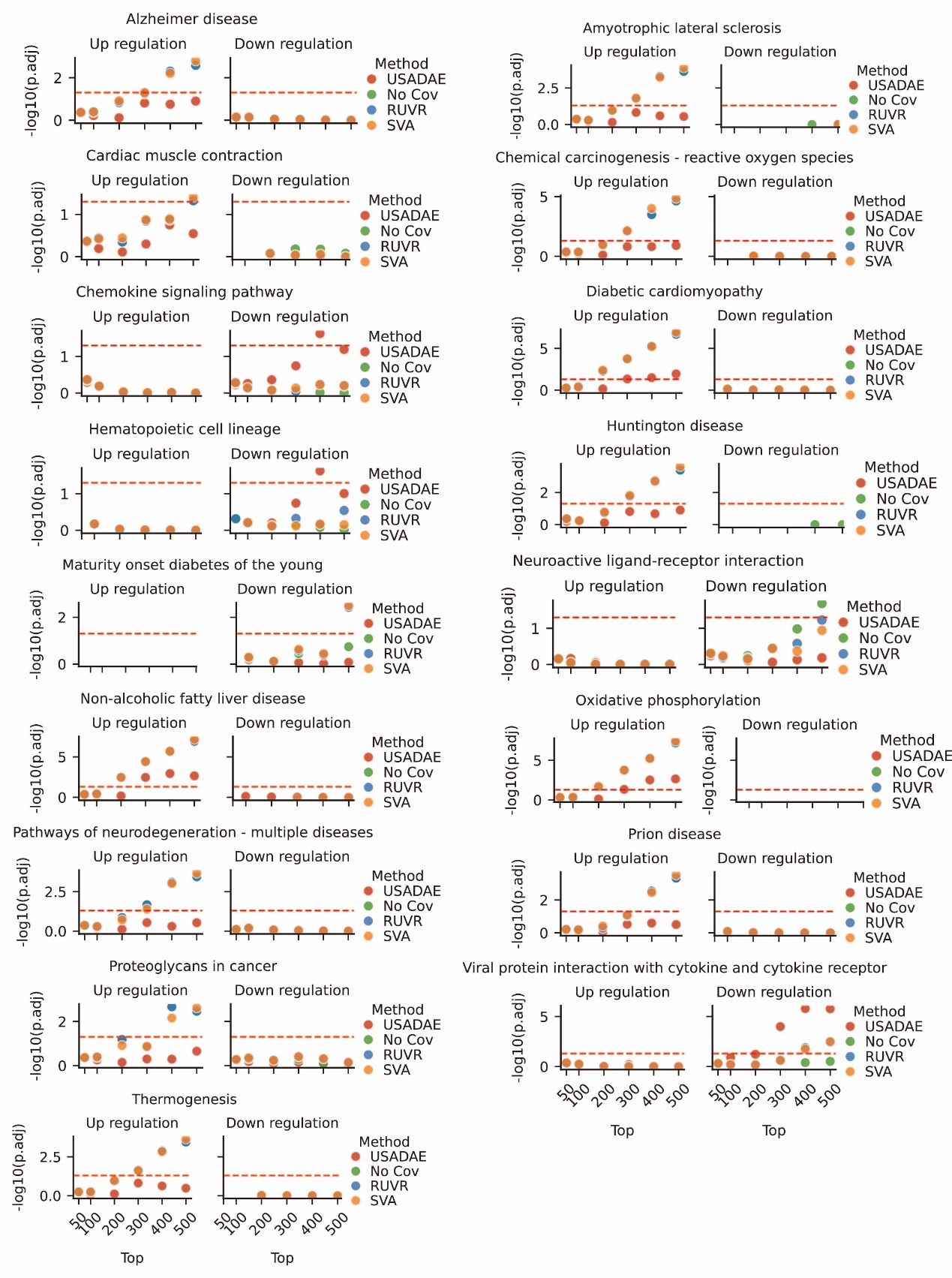


Scatter plots showing the -log10(p-value) of pathway enrichment for the top n differentially expressed genes in pathways associated with breast cancer ER status. The x-axis represents the number of top n genes, and the y-axis displays -log10(p-value). Different colors denote different algorithms used to estimate confounders as covariates.

**Supplementary figure 5**


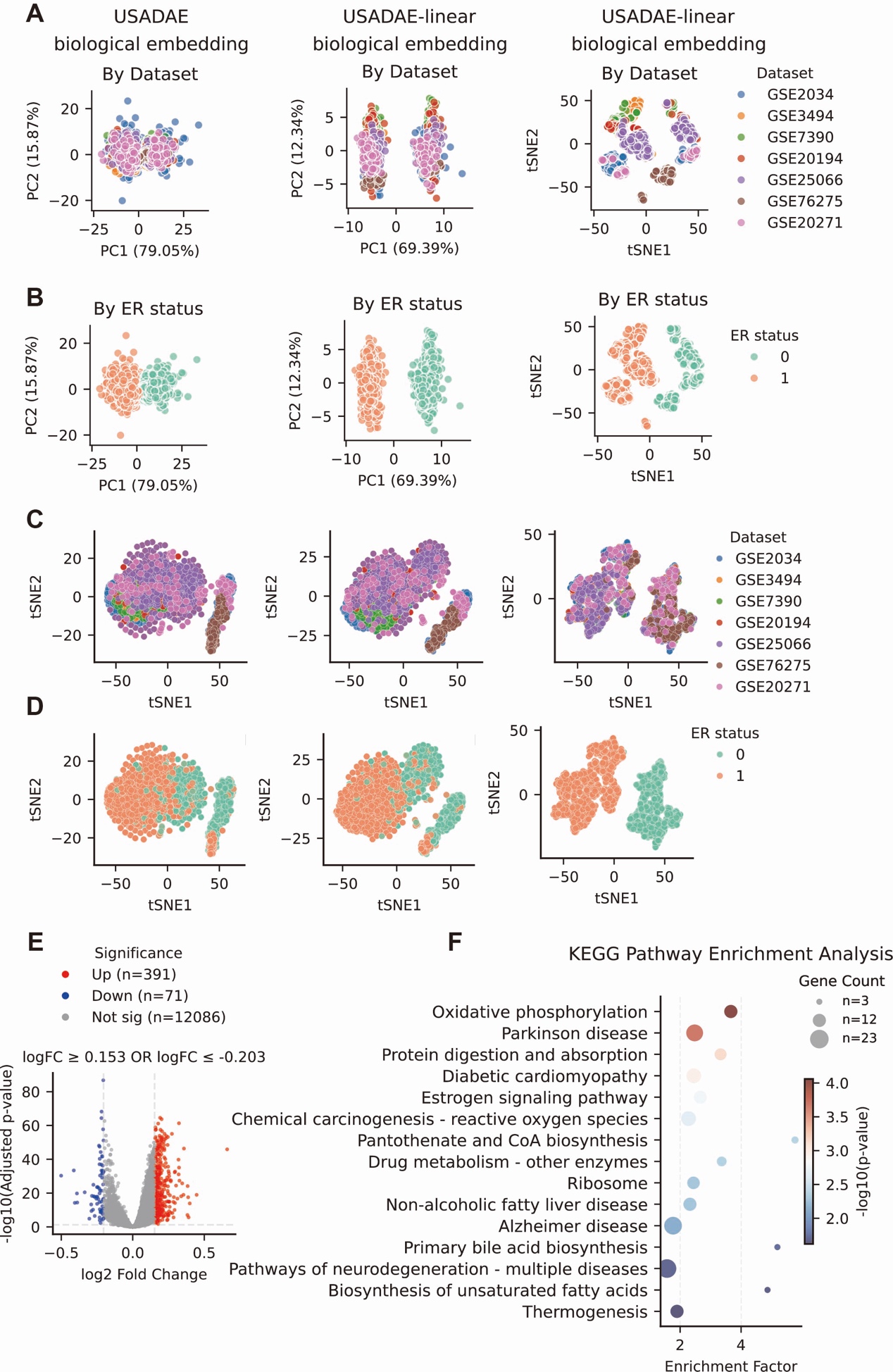


**(A, B)** Dimensionality reduction analysis of biological embeddings. From left to right: PCA plot of USADAE biological embeddings, PCA plot of USADAE-linear biological embeddings, and t-SNE plot of USADAE-linear biological embeddings. (A) Colored by dataset. (B) Colored by ER status. **(C, D)** Comparative visualization of confounder-corrected data using t-SNE. From left to right: RUVR-corrected, SVA-corrected, and USADAE-corrected data. (C) Colored by dataset. (D) Colored by ER status. **(E)** Volcano plot of differentially expressed genes (DEGs) identified by limma after incorporating USADAE-linear estimated covariates. **(F)** Bubble plot of KEGG pathway enrichment of DEGs from (E).

Supplementary figure 6


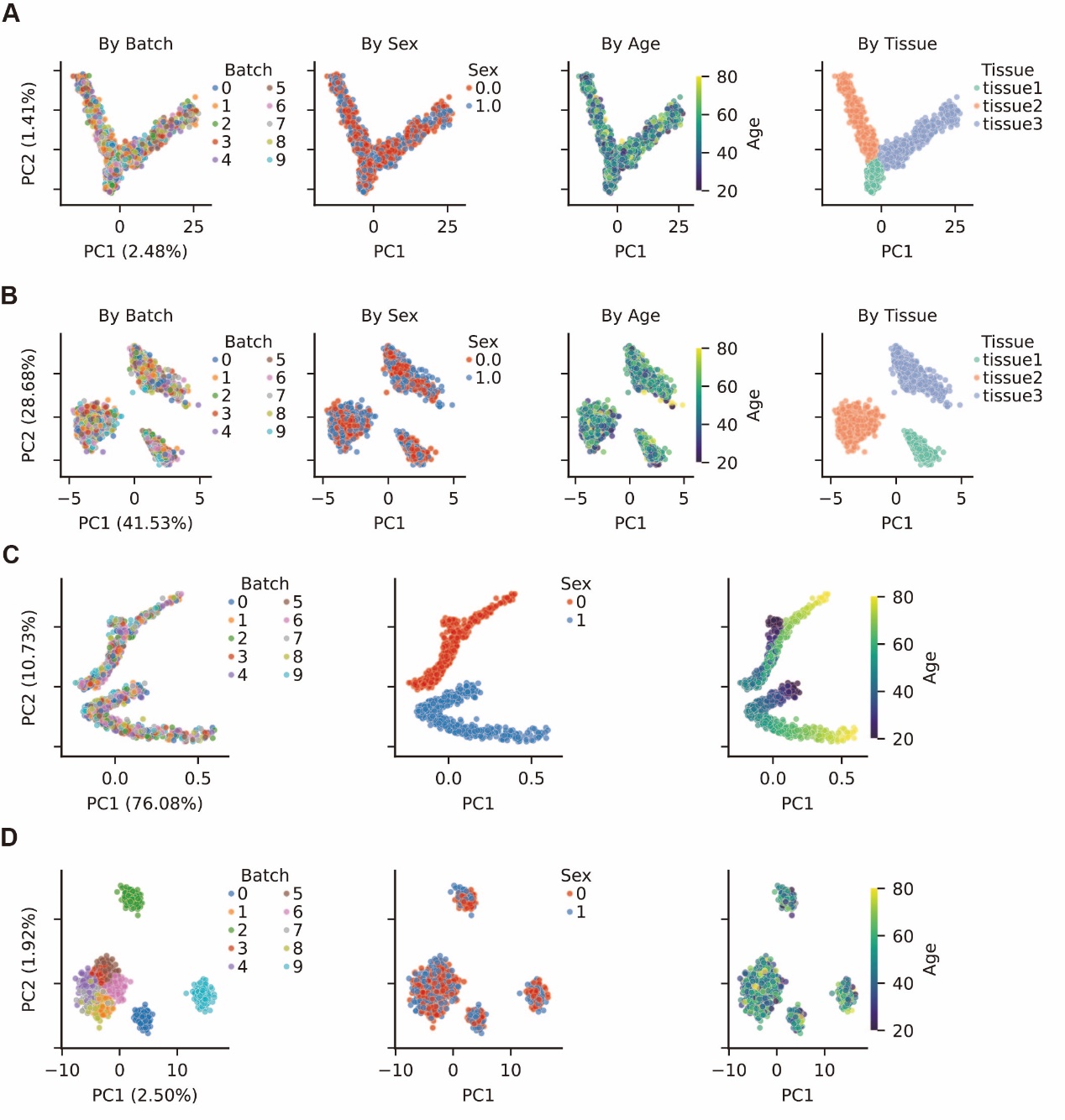


**(A, B)** PCA plot of biological latent and corrected data by USADAE respectively. From left to right, the plots are sequentially colored by batch, sex, age, and group. **(C, D)** PCA plot of estimated confounders and corrected data by PEER from tissue 1

Supplementary figure 7


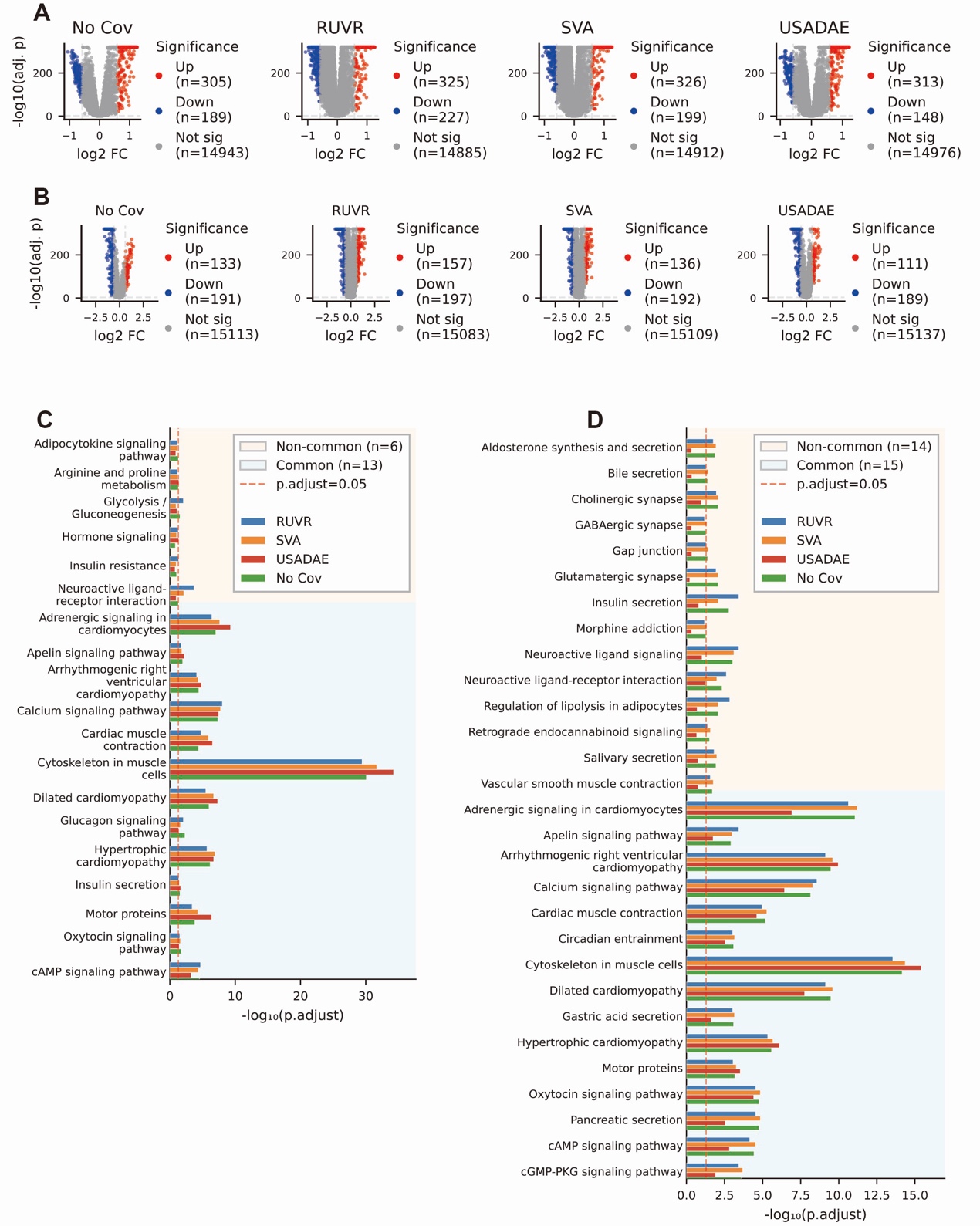


**(A, B)** Volcano plot of differentially expressed genes between muscle and adipose (A) and between muscle and heart (B) when using confounders estimated by No Cov, SVA, RUVR and USADAE as covariates respectively. Horizontal dashed line represents adjusted p-value threshold of 0.05. vertical dashed line represents log2 fold change threshold (No Cov, SVA and RUVR |FC| > 1.5). **(C, D)** KEGG enrichment bar plot of differentially expressed genes in muscle vs. adipose (C) and muscle vs. heart (D). Pathways significantly enriched at least in one method are shown as Non-common. Pathways significantly enriched in all methods are shown as Common.

Supplementary figure 8


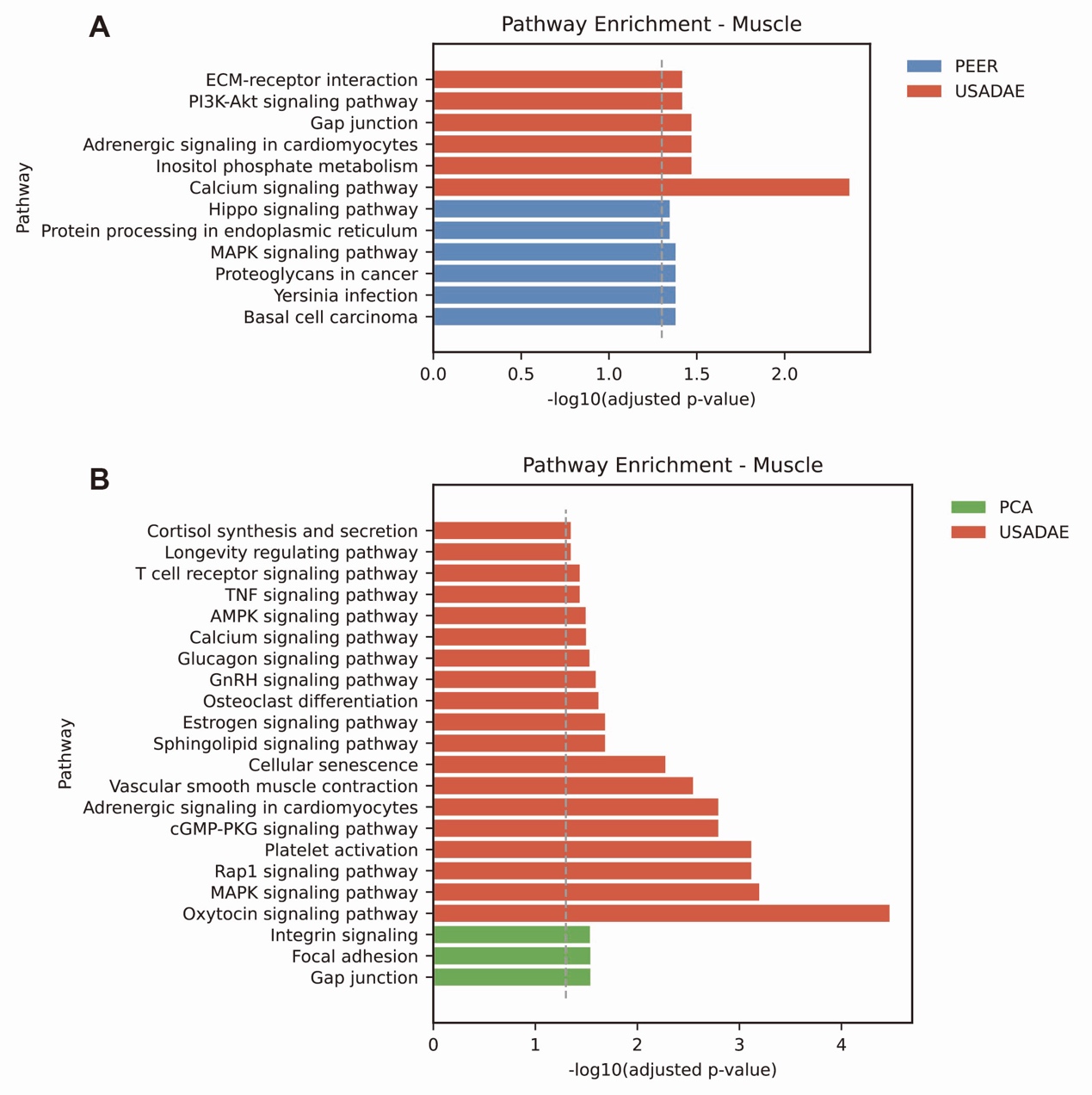


(A) Comparative pathway enrichment analysis between PEER and USADAE methods.​ (B) Comparative pathway enrichment analysis between PCA and USADAE methods.​ Horizontal bar plot showing the ​​-log10(adjusted p-values)​​ of significantly enriched pathways of specific eGenes (no common eGenes) for two covariate adjustment methods.

Supplementary figure 9


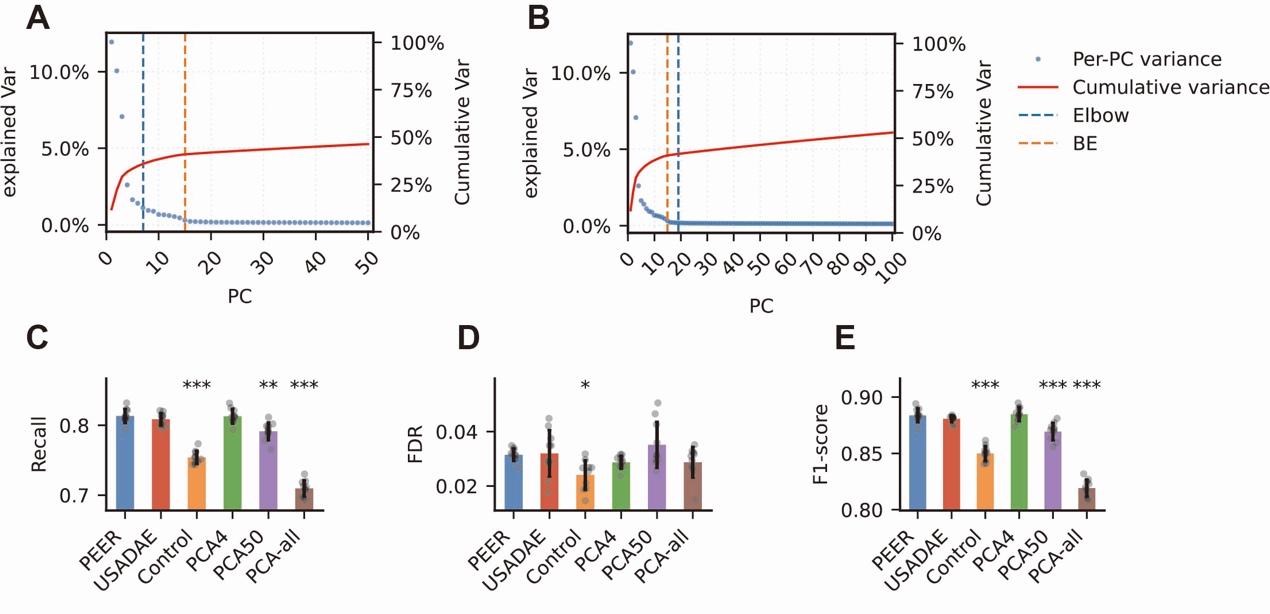


**(A)** Selection of the optimal number of principal components (PCs) from the top 50 PCs using the elbow method and the Buja and Eyuboglu (BE) permutation test. **(B)** Optimal PC selection using all available PCs with the elbow and BE methods. Symbols and line styles are the same as in (A). **(C–E)** Comparison of eGene detection performance when covariates estimated by different methods are used in eQTL mapping. Including too many PCs may capture biological signal and reduce detection power. (C) Recall; (D) false discovery rate (FDR); (E) F1 score. Statistical significance was assessed using paired t-tests with Benjamini–Hochberg correction (*adjusted p < 0.05, **adjusted p < 0.01, ***adjusted p < 0.001).

Supplementary figure 10


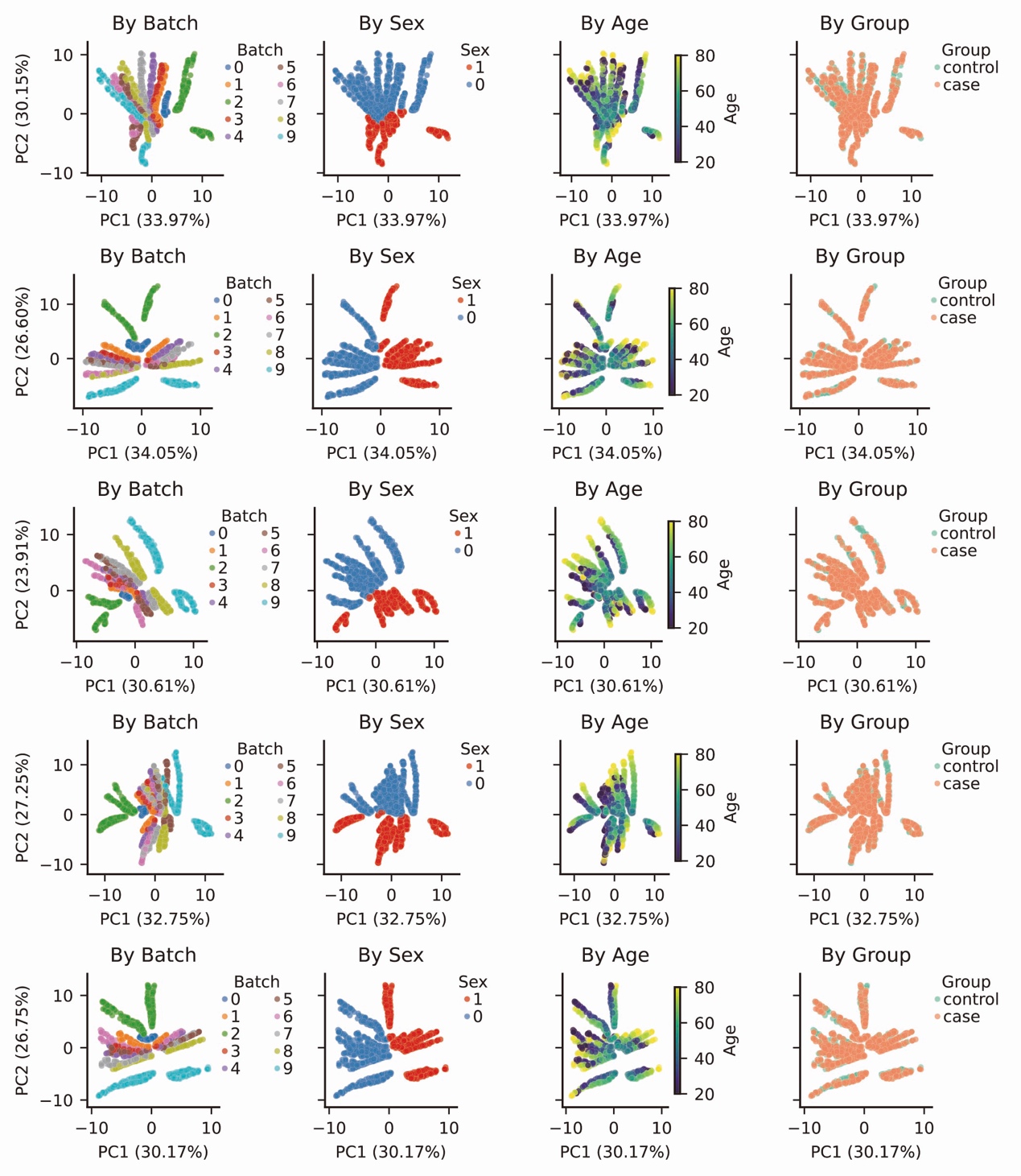


PCA plot of USADAE’s covariates. Prior to model training, the original data were clustered via k-means++ algorithm, with the number of centroids sequentially configured as 500, 1000, 1500, 2000 and 2500 from top to bottom. The raw data used in this analysis were simulated using the same strategy to fig 2, comprising 3000 samples and 15000 genes.

# Supplementary Information

Simulation for differential expression analysis

We developed a comprehensive simulation framework to evaluate differential expression analysis methods under controlled confounding conditions. The simulation protocol consisted of three core components: (1) baseline biological signal generation, (2) nonlinear confounding effect integration, and (3) differential expression introduction, as detailed below.

**1. Baseline Biological Signal Simulation**

Baseline RNA-seq expression counts were generated from a negative binomial distribution to capture realistic mean–variance relationships:

where μ represents baseline mean expression, and ϕ controls dispersion. This parametrization yields dispersed counts consistent with empirical RNA-seq data.

**2. Differential Expression Modeling**

To simulate differential expression, a gene-specific value was assigned to all genes:

For each gene, case-group mean counts were adjusted as , followed by independent negative binomial sampling. This ensures that expression differences arise from underlying distributional changes rather than deterministic scaling. Genes with | | > 0.263 were treaded as true significant differential expression genes.

**3. Confounder Generation**

Three explicit confounders were simulated: Batch (categorical, 10 levels), Age (continuous, 20–80 years), and Sex (binary). Batch effects were modeled as order confounders with 10 levels, each batch was assigned a unique nonlinear expression pattern. For each gene designated as batch-sensitive, a distinct nonlinear function (e.g., sine, tanh, sigmoid, or log-based) was randomly selected to generate batch-specific expression shifts. In addition, cross-batch interaction effects were introduced by applying a second nonlinear transformation to the batch index and adding it as an interaction term. Age effects were generated using nonlinear functions such as sine, quadratic, and sigmoidal transformations of age, with random scaling and noise to capture biological heterogeneity. Sex effects were modeled as baseline offsets modulated by age-dependent nonlinearities:

where (h(A)) represents nonlinear age modulation.

Then all confounder-specific perturbations were multiplicatively applied to the biological signal:

where denotes element-wise multiplication, yielding simulated RNA-seq matrices that jointly encode biological variation and complex, nonlinear confounding effects (Supplementary Fig. 1C-E). This formulation can be viewed as an approximation to mean-level confounder effects, as it induces a corresponding shift in the expected expression levels while allowing additional sample-level variability. All intermediate matrices—including biological signal, confounder effects, and metadata—were saved for benchmarking.

**4. Data Structure and Output**

The simulation generated an expression matrix comprising 3,000 genes and 1,000 samples, with balanced case-control distribution (1,500 cases and 1,500 controls). The associated metadata included batch information with 10 distinct levels, age ranging continuously from 20 to 80 years, and sex distributed equally between males and females. Additionally, ground truth annotations were provided, specifying significantly differentially expressed genes along with their corresponding log2 fold-change values and confounding type classifications. The code for simulation is available at <https://github.com/chenxuya/USADAE>.

Simulated data for eQTL analysis

To evaluate the performance of our method, we simulated multi-tissue expression quantitative trait loci (eQTL) data with muscle genotype data of chromosome 1 obtained from PigGTEx project. The simulation process encompassed three key components: 1) tissue-specific genetic architecture, 2) nonlinear confounding effects, and 3) genotype-expression mapping with biological plausibility.

1. **Tissue-Shared and Tissue-Specific eGene Allocation**

We simulated 2,000 genes across three tissues, where each tissue contained 600-1,000 eGenes following a dynamic allocation algorithm. Let , , and denote the three tissues with eGene counts , , and respectively. The gene sharing structure was determined by solving:

where represents genes shared among three tissues, denotes pairwise shared genes, and indicates tissue-unique eGenes. This allocation ensured biologically realistic overlap patterns while maintaining tissue-specific constraints.

1. **Genetic Effect Simulation**

For each eGene, we modeled cis-regulatory effects through:

**Gene transcription start site (TSS)**: TSSs are assumed to be uniformly distributed across Chromosome 1. Meanwhile, gene lengths follow a normal distribution with a mean of 20 kb and a standard deviation of 5 kb.

**SNP Selection**: Tissue-dependent numbers of cis-SNPs (mean 5-15 per gene) were randomly selected within ±1Mb of the TSS, following a truncated normal distribution (, min=1-3 SNPs vary across genes).

**Effect Size Calculation**: SNP effects () incorporated MAF-dependent nonlinear scaling:

where controlled MAF-dependent decay, and directionality (sign) was randomly assigned1.

**Heritability Control**: Genetic variance () followed a rectified gamma distribution (, vary across tissues) constrained within tissue-specific ranges (0.5%-80%). Effects were scaled to achieve .

1. **Confounder Modeling**

We incorporated three types of nonlinear confounding effects through:

Where batch effects were modeled across ten batches with distinct nonlinear patterns (sigmoid/tanh transformations). Age effects followed biologically plausible functions including U-shaped (), linear decay (), and sigmoidal transitions. Sex effects were simulated as baseline differences with age-dependent interaction terms. Gene-type specificity was enforced: 30% of genes were batch-sensitive, 30% age-related, 20% sex-differential, and the remaining 20% served as null background.

1. **Expression Synthesis**

Final expression values integrated genetic and confounding effects:

where denotes element-wise multiplication, and tissue-specific noise variance () ranged 0.8-1.2. Genotype data for 10,000 SNPs was simulated with MAF > 1%, and sample IDs were replicated across tissues to mimic paired multi-tissue designs. Code availability and parameter details are provided in Supplementary Materials.

Evaluation metrics

**Classification metrics**

1. **Area Under ROC Curve (AUC):**

Where denotes the classification threshold and ROC (·) represents the Receiver Operating Characteristic curve.

1. **Accuracy**:

Where , , , and denote True Positives, True Negatives, False Positives, and False Negatives, respectively.

1. **F1-Score**:

With Precision () and Recall () defined as:

1. **Specificity**:
2. **FDR**:
3. **Macro-Averaged Metrics** (for multiclass problems):

- Where is the number of classes and subscript denotes class-specific metric.

**Regression metric**

**Coefficient of determination ()**:

Where = true value, = predicted value, and = mean of true values.

# Reference

1. Schoech, A. P. *et al.* Quantification of frequency-dependent genetic architectures in 25 UK Biobank traits reveals action of negative selection. *Nat Commun* **10**, 790 (2019).
